# Dual Anticoagulation With Warfarin and Apixaban: A Case Report

**DOI:** 10.7759/cureus.29093

**Published:** 2022-09-12

**Authors:** Kristen Parker, Paula Yonosko, Deon Pilkington

**Affiliations:** 1 Pharmacy Services, Banner Health, Phoenix, USA; 2 Pharmacy, Banner Baywood Medical Center, Phoenix, USA

**Keywords:** pulmonary embolism (pe), deep vein thrombosis (dvt), dual anticoagulation, apixaban, warfarin

## Abstract

We report the case of a female with recurrent venous thromboembolisms (VTEs) despite being appropriately anticoagulated. Different anticoagulants were trialed and all led to treatment failure. As a result, the patient was started on dual anticoagulation with warfarin and apixaban. There were no recurrences of clots while the patient was adherent to dual anticoagulation. This case report adds to current literature demonstrating the efficacy of dual anticoagulation in patients with recurrent VTE who fail single-agent therapy. It also demonstrates the need for additional studies that evaluate the utility of dual anticoagulants as a treatment modality for patients failing monotherapy.

## Introduction

Venous thromboembolism (VTE), a disorder that includes deep venous thromboembolisms (DVT) and pulmonary embolisms (PE), is associated with significant morbidity and mortality. Although the precise number of people affected by VTEs is unknown, it is estimated that as many as 900,000 people are affected per year in the United States [1]. VTEs can develop in anyone, however, certain patient populations are at higher risk. Patients who have a past medical history of previous VTE, cancer, or thrombophilia are at higher risk of VTE development. Approximately five to eight percent of the United States population has an inherited thrombophilia in which a genetic defect leads to an increased risk for thrombosis [1]. Anticoagulation therapy is the mainstay of therapy for the treatment and prevention of VTEs in patients at high risk. In most cases, a single anticoagulant is sufficient to prevent VTEs [2]. However, in rare instances, it is not [3,4]. We present a case of a young female with an extensive VTE history who required dual anticoagulation therapy to prevent clot recurrence.

## Case presentation

A 44-year-old Caucasian female was referred to a pharmacist-managed anticoagulation clinic. The patient was originally initiated on anticoagulation therapy at the age of 15 due to a history of antiphospholipid antibody syndrome (APLS; lupus anticoagulant positive), Factor V Leiden deficiency (unknown whether homozygous or heterozygous), and recurrent DVTs and PEs. The patient reported greater than 25 PEs and 50 DVTs. Her family history was not significant on her maternal side for a thromboembolic disorder, and she was unaware of her family history on her paternal side. Pertinent social history included being a previous tobacco smoker of one-half a pack per day. It is unknown how long the patient was a smoker, but she reported quitting over 30 years ago. Attempts in the past to have an inferior vena cava (IVC) filter placed were unsuccessful due to the presence of micro clots and 21 stents. For pharmacologic management, the patient had tried and failed numerous monotherapy anticoagulants and combination anticoagulant and antiplatelet regimens, despite being adherent. As monotherapy, the patient failed warfarin with a therapeutic international normalized ratio (INR) goal ranging, at various times, from 2 to 5, as well as dabigatran, apixaban, and fondaparinux, based on having recurrent clots while taking these agents. The patient’s previous combination therapies included warfarin with clopidogrel, warfarin with aspirin 325 milligrams (mg), apixaban with clopidogrel, fondaparinux with clopidogrel, and fondaparinux with clopidogrel and aspirin 325 mg. The patient also had a history of heparin-induced thrombocytopenia (HIT) with both heparin and enoxaparin, further limiting anticoagulant therapy options.

Dual anticoagulation was initiated when the patient presented to the emergency department (ED) for left groin and right calf pain, which had been present for two weeks. The patient was being managed at the time on warfarin monotherapy with a therapeutic INR goal of 3.5. The patient’s INR two weeks prior to the ED visit was therapeutic at 3.5. The patient’s last incidence of a clot was about eight months prior. Upon arrival to the ED, a bilateral lower extremity ultrasound indicated the presence of multiple clots with at least four being occlusive. Her INR was found to be 2, and she reported good adherence to warfarin at the time. Due to the patient’s extensive clot history, prior treatment failures, and history of HIT on both heparin and enoxaparin, dual anticoagulation was recommended per inpatient hematology. Dual anticoagulation was initiated with apixaban 2.5 mg twice daily and warfarin with a therapeutic INR goal of 2 to 3. After initiation, dual anticoagulation was successful, with no new VTEs for two years. During this two-year time period, the patient had a time in therapeutic range (TTR) of 78.2% based on 28 INR tests, covering 742 days per the Rosendaal TTR calculation [5]. The patient also did not experience any significant bleeding events during this time.

Due to insurance issues and the patient being unable to fill apixaban, the patient went off apixaban for two weeks and was managed on warfarin monotherapy. During this time, the patient was admitted for a non-occlusive VTE in the left common femoral vein. At the time of admission, INR was found to be supratherapeutic at 5.4, despite new clot development. Her INR in the clinic three weeks prior to this had been 2.2. Following this, dual anticoagulation with warfarin and apixaban was restarted. The goal INR of warfarin remained the same at 2 to 3, however, the dose of apixaban was increased from 2.5 mg twice daily to 5 mg twice daily at the discretion of hematology. At the time of writing this, the patient had not had a reoccurrence of a clot or any significant bleeding events on this regimen. As the latest clot occurred when the patient was off apixaban therapy, it further justified the need for dual anticoagulation in this patient and, potentially, other patients with a similar thrombophilia history.

## Discussion

This case demonstrates the utility of dual anticoagulation with apixaban and warfarin in a patient with an extensive history of recurrent clots despite the use of monotherapy anticoagulants and combination anticoagulant and antiplatelet treatment regimens. There is no mention of combining a direct-acting oral anticoagulant (DOAC) with warfarin for VTE in treatment consensus guidelines. However, per the literature review, there are several case reports on other DOACs used with warfarin. One case report demonstrated the efficacy of rivaroxaban 15 mg twice daily in combination with warfarin with a therapeutic goal INR range of 2-3 in a patient with no known thrombophilias [3]. Two other case reports presented patients with diagnoses of cancer successfully managed on dual anticoagulation with dalteparin in combination with warfarin with a therapeutic goal INR range of 2-3 [4]. To date, no case reports have been published demonstrating dual anticoagulation with apixaban and warfarin.

It is important to acknowledge that the information gathered for this report is based on an extensive chart review and discussion with the patient. The chart review was limited to the records accessible within our health system and dated back to when the patient was 28 years of age. Inpatient and outpatient records that originated outside of our organization were not accessible for review. In addition, adherence was assumed based on the patient requesting medication refills from our clinic on time and based on time in the therapeutic range. Although limitations in the data available for review exist, the information that we have reported has been validated, as we have been actively following and managing the patient’s warfarin therapy for over two years. This information also adds to the minimal literature that exists regarding the combination use of DOACs and warfarin.

## Conclusions

In summary, this case demonstrates safe and effective dual anticoagulation therapy with warfarin and apixaban for the prevention of VTE recurrence in a patient with an extensive clotting history. Dual anticoagulation should not be routinely used due to the increased risk of adverse events such as bleeding. However, in patients such as this, with an extensive clot history and failure of anticoagulation monotherapy, the benefits of dual anticoagulation may outweigh the risks. This is the first case to demonstrate efficacy with the combination of apixaban and warfarin. Previous studies have also demonstrated success with the combination of warfarin and rivaroxaban and warfarin and dalteparin.
